# Factors associated with intentions to quit tobacco use in Lebanon: A cross-sectional survey

**DOI:** 10.1016/j.pmedr.2023.102572

**Published:** 2023-12-22

**Authors:** Dina Farran, Ruba Abla, Rima Nakkash, Niveen Abu Rmeileh, Mohammed Jawad, Yousef Khader, Aya Mostafa, Ramzi G. Salloum, Ali Chalak

**Affiliations:** a Department of Psychosis Studies, Institute of Psychiatry, Psychology and Neuroscience, King’s College London, London, UK; b Department of Health Promotion and Community Health, Faculty of Health Sciences, American University of Beirut, Lebanon; c Institute of Community and Public Health, Birzeit University, Birzeit, Palestine; d Public Health Policy Evaluation Unit, Imperial College London School of Public Health, London, UK; e Department of Community Medicine, Public Health and Family Medicine, Faculty of Medicine, Jordan University of Science and Technology, Irbid, Jordan; f Department of Community, Environmental and Occupational Medicine, Faculty of Medicine, Ain Shams University, Cairo, Egypt; g Department of Health Outcomes & Biomedical Informatics, University of Florida College of Medicine, Gainesville, FL, USA; h Department of Agriculture, Faculty of Agricultural and Food Sciences, American University of Beirut, Beirut, Lebanon; i Global and Community Health Department, George Mason University, VA, USA

**Keywords:** Waterpipe tobacco use, Cigarette smoking, Intention to quit smoking, Quitting attempt, Smoking cessation

## Abstract

**Introduction:**

The prevalence of tobacco smoking in Lebanon is among the highest globally. This study aims to determine past attempts to quit smoking among adults and identify factors associated with intentions to quit.

**Methods:**

A nationally representative telephone survey was conducted between June and August 2022. Eligibility criteria included people aged >=18 years residing in Lebanon. The questionnaire was divided into three components: socio-demographic characteristics, cigarette and waterpipe tobacco use behaviours. Binary logistic regression was used to examine factors associated with intention to quit cigarette and waterpipe tobacco use.

**Results:**

A total of 2003 respondents were included in the study. The prevalence of any tobacco product use was 41%, the prevalence of current cigarette smoking was 41% and the prevalence of current waterpipe tobacco use was 20%. Approximately 24% of adults who smoke cigarettes and 26% of those who use waterpipe tobacco had previous quit attempts mainly due to health concerns. Intentions to quit smoking within the next 6 months were reported among 12% of survey respondents. Among adults who smoke, past quit attempts increased the likelihood of intentions to quit cigarette smoking by 5-fold (OR: 5.11; 95% CI: 1.80–14.47, p = 0.002) and waterpipe tobacco use by 7-fold (OR: 6.98, 95% CI: 2.63–18.51, p = <0.001). Age and income were associated with intentions to quit cigarette but not waterpipe tobacco use.

**Conclusion:**

Intention to quit smoking was strongly associated with past quitting attempts. Understanding factors associated with intentions to quit can help inform the development of context specific smoking cessation interventions.

## 1 Introduction

Tobacco smoking is one of the major public health threats globally responsible for more than 8 million deaths annually (Tobacco World Health Organization, xxxx). Despite the known health risks associated with smoking, more than 1.3 billion people continue to use tobacco with 80% of them living in low- and middle-income countries (Tobacco World Health Organization, xxxx). The Global Adult Tobacco Survey conducted in 28 countries between 2008 and 2016 reported that the prevalence of tobacco smoking among people aged above 15 years was 23% and that 43% of those who smoke had made an attempt to quit smoking in the preceding year (Ahluwalia et al., 2018). The same source reported that the prevalence of past year quit attempt was 41% in Egypt and 38% in Qatar (Ahluwalia et al., 2018). In Jordan, 45% of people who smoke have made a quitting attempt during the past 12 months in 2019 (Survey, 2023).

Lebanon is among the countries of the Eastern Mediterranean Region where both cigarette and waterpipe tobacco use are highly prevalent (World Health Organization, xxxx). A nationally representative household survey conducted in 2019 with 1680 respondents in Lebanon showed that the prevalence of cigarette smoking was 49% among men and 22% among women (Nakkash et al., 2022). The prevalence of waterpipe tobacco use was 33% and 46% among men and women respectively (Nakkash et al., 2022). The world health organisation STEPwise approach to noncommunicable diseases and risk factors surveillance survey carried out in Lebanon between 2016 and 2017 reported that out of every 10 adults who smoked, 2 had quit attempts and 4 were advised by a healthcare professional to quit in the preceding 12 months (World Health Organization. Lebanon STEPS survey, 2017).

Several factors can contribute to differences in quit attempts including tobacco control policies, attitudes towards smoking (e.g., peer pressure, social norms), and the availability of resources for smoking cessation (i.e., cessation programs and medications). Much of the available evidence on tobacco treatment interventions comes from high-income countries and has not been thoroughly tested and adjusted to low-income contexts (West et al., 2015, Piné-Abata et al., 2013). The literature supports the efficacy and affordability of such interventions yet recognizes the substantial practical and financial barriers to their implementation in low-income settings (West et al., 2015).

Tobacco control policies in low- and middle-income countries may be less stringent or poorly enforced (Yong et al., 2022, Pourtau et al., 2019, Echer and Barreto, 2008, Twyman et al., 2014, Minichino et al., 2013). Lebanon has a weak tobacco policy framework, and despite high smoking prevalence, there is a lack of public resources for implementing smoking cessation support services and smoking cessation policies (Maziak et al., 2014, Bacha et al., 2018).

Although many studies have determined the prevalence of cigarette and waterpipe tobacco use as well as factors associated with quit attempts or intentions to quit smoking worldwide, there is very limited information on these topics from the Eastern Mediterranean Region, and particularly from Lebanon (Marques-Vidal et al., 2011, Thyrian et al., 2008, Mostafa, 2020, Mostafa et al., 2018). This may be attributed to the social norms towards smoking that may be more accepting in the region.

In response to this knowledge gap, this study aimed to: (i) determine pattern of tobacco smoking and tobacco smoking cessation among men and women; and (ii) determine factors associated with intentions to quit cigarette and waterpipe tobacco use. We hypothesis that (i) the prevalence of cigarette and waterpipe smoking is higher among men than women, (ii) patterns of cigarette and waterpipe smoking cessation differ significantly between men and women and (iii) intentions to quit cigarette and waterpipe smoking are significantly likelier with past quitting attempts.

## 2 Methods

### 2.1 Study design and data collection

A nationally representative telephone survey was conducted in Lebanon between June and August 2022. The eligibility criteria included men and women aged 18 years and older residing in Lebanon regardless of their nationality. The sample included respondents from all eight governorates of Lebanon. A random digital selection of phone numbers in each governorate was applied with a continuous monitoring of the areas (cities, towns, villages) covered to ensure a large geographical representativeness. A target sample size of 1,940 was sought. To that end, a total of 8,071 calls were made with 6,068 resulting in no participation for any the following reasons: no answer, number unavailable, wrong number, refusal, or respondent not eligible. An actual sample of 2,003 respondents was achieved, of whom 1,005 (50.2%) were women and 998 (49.8%) were men.

After eligibility screening, potential respondents were provided with information about the study and asked to provide verbal consent to participate. Data were collected using a structured Arabic questionnaire. Interviews lasted for a maximum of 30 min. Data collectors were trained on the ethics of data collection, consenting, sampling methodology and on the survey questions. The training for data collectors included a review of each questionnaire item, followed by practice sessions which involved roleplay.

### 2.2 Measures

The questionnaire was divided into three main parts. The first part included self-identified demographic characteristics such as sex, age, marital status, education, income, and nationality. The second part addressed cigarette and waterpipe tobacco use. Adults who reported cigarette or waterpipe tobacco use were asked about their smoking frequency, years of smoking and the effect of the increase in price of tobacco products on their consumption levels. A series of questions related to the smoking cessation experience based on the Transtheoretical Model of behaviour change, which breaks down the process of quitting smoking into five stages—precontemplation, contemplation, preparation, action, and maintenance—were included (Prochaska et al., 1997). This includes questions on thinking of quitting cigarette and waterpipe tobacco use within the next 6 months, attempts to quit tobacco smoking during the past 12 months, reasons for quitting, and duration of the abstinence period.

### 2.3 Statistical analysis

Descriptive statistics were generated to summarise respondents’ socio-demographic and relevant characteristics. Categorical variables were presented as counts and percentages. Chi-squared test was used to compare the prevalence of tobacco use as well as intentions and behaviors related to quitting cigarette and waterpipe tobacco use among survey respondents stratified by sex group. Binary logistic regression examined factors associated with the intention to quit cigarette and waterpipe tobacco use in separate models. The independent variables tested in these models included previous quitting attempts in general, quitting attempts due to lack of affordability, quitting attempts due to health concerns, household income, education, marital status and age (Mostafa, 2020, Mostafa et al., 2018, Prochaska et al., 1997, Maralani, 2014, Ruokolainen et al., 2021, Holm et al., 2017, Reid et al., 2010, Hiscock et al., 2015, Nasser et al., 2020, Akl et al., 2015, Salloum et al., 2019, Almeda and Gómez-Gómez, 2022, Feng et al., 2010, Hyland et al., 2006). Odd ratios (OR) and their 95% Confidence intervals (CI) were estimated from the models. A p-value of less than 0.05 was considered statistically significant. Statistical analyses were performed using R Studio and Stata statistical software V.16.

### Ethical approval

This study adheres to the ethical principles outlined by the American University of Beirut, emphasizing the importance of ensuring the safety, privacy, and rights of human subjects involved in the research. The research protocol received approval from the Institutional Review Board, and all procedures were conducted in accordance with their guidelines.”.

## 3 Results

### 3.1 Participants’ characteristics

Table 1 shows the demographic characteristics of the respondents stratified by sex group. Less than half (43.2%) of respondents were aged between 30 and 49, 66.5% were married and 42.8% had a university degree. Around 32.9% had no individual income and 37.0% had income below 5,000,000 Lebanese Pounds (160 USD).

**Table 1:** Total and by sex demographic characteristics of Lebanese survey respondents (n = 2003).

**Variable**	**Total** **n = 2003**	**Men** **n = 998**	**Women** **n = 1005**
**Age, in years**			
18 – 29	476 (23.8)	215 (21.5)	261 (26.0)
30 – 49	866 (43.2)	442 (44.3)	424 (42.2)
50 – 64	535 (26.7)	303 (30.4)	232 (23.1)
≥65	126 (6.3)	38 (3.8)	88 (8.8)
**Marital status (Married)**	1321 (66.5)	686 (69.3)	635 (63.7)
**Education**			
Primary school or less	275 (13.8)	124 (12.5)	151 (15.2)
Intermediate	373 (18.8)	196 (19.8)	177 (17.8)
Secondary	488 (24.6)	255 (25.7)	233 (23.4)
University	850 (42.8)	417 (42.0)	433 (43.6)
**Employment status (employed)**	982 (49.0)	645 (64.6)	337 (33.5)
**Individual Income, in Lebanese Pounds**			
<3000 000	412 (20.6)	237 (23.7)	175 (17.4)
>= 3000 000 to < 5 000 000	329 (16.4)	201 (20.1)	128 (12.7)
>= 5000 000 to < 10 000 000	267 (13.3)	202 (20.2)	65 (6.5)
>= 10 000 000	103 (5.1)	79 (7.9)	24 (2.4)
No income	659 (32.9)	156 (15.6)	503 (50.0)
**Household income, in Lebanese Pounds**			
<3000 000	367 (18.3)	163 (16.3)	204 (20.3)
>= 3000 000 to < 5 000 000	456 (22.8)	225 (22.5)	231 (23.0)
>= 5000 000 to < 10 000 000	580 (28.9)	307 (30.7)	273 (27.2)
>= 10 000 000	186 (9.2)	114 (11.4)	72 (7.2)
No income	48 (2.4)	28 (2.8)	20 (2.0)
**Income currency**			
In Lebanese pounds	1037 (77.2)	657 (78.0)	380 (75.7)
Partially in Lebanese Dollar equivalent	12 (0.9)	8 (1.0)	4 (0.8)
Fully in Lollars	5 (0.4)	1 (0.1)	4 (0.8)
Partially in Dollars	60 (4.5)	45 (5.3)	15 (3.0)
Fully in US Dollars	52 (3.9)	40 (4.8)	12 (2.4)

Table 2 shows the prevalence of tobacco smoking among men and women respondents. Approximately 41.4% of respondents (46.4% of men and 36.5% of women) used tobacco products. The rate of cigarette smoking was 22.0% with significantly more men than women reporting current cigarette smoking (29.7% vs 14.3%, p < 0.001). The prevalence of current waterpipe tobacco use was 19.5%; with a higher prevalence of waterpipe tobacco use noted in women compared to men (22.6% vs 16.4%, p < 0.001).

**Table 2:** Total and by sex cigarette and waterpipe tobacco use among Lebanese survey respondents (n = 2003).

**Variable**	**Total** **n = 2003**	**Men** **n = 998**	**Women** **n = 1005**	**P value**
No current tobacco use	1173 (58.6)	535 (53.6)	638 (63.5)	<0.001
Any current tobacco use	830 (41.4)	463 (46.4)	367 (36.5)	<0.001
Current cigarette smoking	440 (22.0)	296 (29.7)	144 (14.3)	<0.001
Current waterpipe tobacco use	391 (19.5)	164 (16.4)	227 (22.6)	<0.001
Other current tobacco product(s) use	30 (1.5)	22 (2.2)	8 (0.8)	0.016

### 3.2 Cigarette and waterpipe tobacco cessation

Table 3 summarises the intentions to quit smoking, past quit attempts, reasons for quitting and the use of other tobacco products or any forms of help when quitting. It also presents the self-reported effects of tobacco price increases on the purchase of cigarettes and waterpipe tobacco.

**Table 3:** Total and by sex intentions and behaviors related to quitting cigarettes and waterpipe tobacco use among Lebanese survey respondents.

**Variable**	**Total**	**Men**	**Women**	**P value**
A. **Cigarette smoking**				
**n**	440 (22.0)	296 (29.7)	144 (14.3)	
**Thinking of quitting**	51 (11.6)	35 (11.8)	16 (11.1)	0.952
**Tried quitting**	107 (24.3)	75 (25.3)	32 (22.2)	0.551
**Reasons for quitting**				
Worried about health consequences	79 (73.8)	58 (77.3)	21 (65.6)	0.307
Cannot afford smoking	14 (13.1)	9 (12.0)	5 (15.6)	0.845
Worried about catching COVID-19	2 (1.9)	0 (0.0)	2 (6.2)	0.160
Worried about COVID-19 complications	2 (1.9)	2 (2.7)	0 (0.0)	0.878
Difficult to access cessation resources	1 (0.9)	0 (0.0)	1 (3.1)	0.659
Other reasons	20 (18.7)	12 (16.0)	8 (25.0)	0.411
**Used other tobacco products when quitting**	8 (7.5)	6 (8.0)	2 (6.2)	1.00
**Received help when quitting**	5 (4.7)	4 (5.3)	1 (3.1)	1.00
**Effect of price increase on cigarette purchases**				0.678
Increased	48 (11.0)	34 (11.5)	14 (9.8)	
Decreased	182 (41.6)	125 (42.4)	57 (39.9)	
Was not affected	208 (47.5)	136 (46.1)	72 (50.3)	
B. **Waterpipe smoking**				
**n**	391 (19.5)	164 (16.4)	227 (22.6)	
**Thinking of quitting**	47 (12.0)	20 (12.2)	27 (11.9)	1.000
**Tried quitting**	100 (25.6)	43 (26.2)	57 (25.1)	0.896
**Reasons for quitting**				
Worried about health consequences	47 (47.0)	25 (58.1)	22 (38.6)	0.083
Cannot afford smoking	14 (14.0)	4 (9.3)	10 (17.5)	0.376
Worried about catching COVID-19	14 (14.0)	7 (16.3)	7 (12.3)	0.780
Worried about COVID-19 complications	11 (11.0)	3 (7.0)	8 (14.0)	0.427
Difficulty to access cessation resources	2 (2.0)	1 (2.3)	1 (1.8)	1.00
Other reasons	27 (27.0)	10 (23.3)	17 (29.8)	0.614
**Used other tobacco products when quitting**	4 (4.0)	1 (2.3)	3 (5.3)	0.821
**Received help when quitting**	0 (0.0)	0 (0.0)	0 (0.0)	NA
**Effect of price increase on waterpipe purchases**				0.422
Increased	14 (4.4)	4 (3.0)	10 (5.4)	
Decreased	142 (44.8)	57 (42.9)	85 (46.2)	
Was not affected	161 (50.8)	72 (54.1)	89 (48.4)	

Approximately 24.3% of adults (25.3% of men and 22.2% of women) who currently smoked cigarettes made a previous quit attempt. Of those, 74% reported that they were worried about their health, 13% were not able to afford it anymore due to the price increase, 4% were worried about COVID-19 and its complications and 19% had other reasons; 12% of adults who currently smoked cigarettes reported that they were thinking of quitting cigarette within the next 6 months.

Similarly, 26% of adults who used waterpipe tobacco had previously made a quit attempt. The main reasons for quitting were concerns about their health in general (47%), worries about COVID-19 and its complications (25%) and other reasons (27%); 12% of adults who currently used waterpipe tobacco reported having intentions to quit within the next 6 months.

As for the effect of price increase on the purchase of tobacco, 42% of adults who smoked cigarettes reported a decrease in cigarette purchases, 48% reported no effect and 11% reported an increase in cigarette purchases. Similarly, 45% of adults who used waterpipe tobacco reported a decrease in waterpipe tobacco purchases, 51% reported no effect and 4% reported an increase in waterpipe tobacco purchases after the inflation.

### 3.3 Factors associated with the intent to quit cigarettes and waterpipe tobacco use

Table 4 presents the results of a binary logistic regression analysis to examine the association of factors, such as past quit attempts, income, education, marital status, and age, with the intention to quit cigarettes and waterpipe tobacco use.

**Table 4:** Multivariable analysis of quitting attempts and sociodemographic factors associated with intentions to quit cigarette and waterpipe tobacco use among Lebanese respondents.

	**Intention to quit smoking within the next 6 months** **OR (95% CI)**	
	**Cigarette**	**Waterpipe**
**Past quitting attempt**		
No/Don't know/No answer	Reference	Reference
Yes	5.11 (1.80–14.47)	6.98 (2.63–18.51)
**Past quitting attempt due to lack of affordability**		
No/Don't know/No answer	Reference	Reference
Yes	1.71 (0.48–6.07)	2.37 (0.65–8.72)
**Past quitting attempt due to health concerns**		
No/Don't know/No answer	Reference	Reference
Yes	0.98 (0.35–2.76)	0.37 (0.13–1.08)
**Household income**		
<LBP 5,000,000	Reference	Reference
>=LBP 5,000,000	0.41 (0.19–0.86)	0.53 (0.25–1.12)
**Marital status**		
Married	0.93 (0.44–1.93)	1.61 (0.73–3.55)
Other	Reference	Reference
**Education**		
Primary/intermediate	Reference	Reference
Secondary	0.99 (0.44–2.24)	1.20 (0.50–2.89)
University	1.59 (0.71–3.57)	0.85 (0.36–2.03)
**Age group**		
18–34	Reference	Reference
35–49	2.09 (1.06–4.10)	1.42 (0.63–3.17)
50 or more	Omitted	1.39 (0.52–3.69)

In the cigarettes model, adults who reported previous quit attempts were five times higher odds to report intentions to quit smoking within the next 6 months, compared to those with no previous attempts (OR: 5.11; 95% CI: 1.80–14.47, p = 0.002). When further stratified by reasons for quitting, we found no association between previous quit attempts due to unaffordability with intentions to quit and between previous quit attempts due to health concerns with intentions to quit within the next 6 months. Adults with a household income of more than 5 million Lebanese Pounds (160 USD) were less likely to have intentions to quit cigarette smoking compared to those earning less than 5 million Pounds (OR: 0.41; 95% CI: 0.19–0.86, p = 0.019). The odds of thinking of quitting cigarette smoking was two times higher in adults aged between 35 and 49 compared to other age groups (OR: 2.1; 95% CI: 1.1–4.1, p = 0.033). Education and marital status showed no association with the intention to quit cigarette smoking.

In the waterpipe tobacco model, past quit attempt(s) increased the likelihood of reporting intentions to quit waterpipe tobacco use by 7-fold (OR: 6.98, 95% CI: 2.63–18.51, p < 0.001). When stratified by reasons for quitting, no association was found between previous quit attempts due to health concerns or affordability with the intention to quit waterpipe tobacco use within the next 6 months. Additionally, no association was found between age, marital status, education, or household income with the intentions to quit waterpipe tobacco use.

## 4 Discussion

This study provides new insights on the intentions to quit smoking among adults in Lebanon. The prevalence of cigarette and waterpipe tobacco use was 22% and 20% respectively with cigarette smoking being more common among men and waterpipe tobacco use more common among women. Around 24% of adults who smoked cigarettes and 26% of those who used waterpipe tobacco had previous quitting attempts mainly due to health concerns. Intentions to quit smoking within the next 6 months were reported among 12% of those who smoked. The odds of having intentions to quit cigarette and waterpipe tobacco use were five and seven times respectively higher among adults who had previous quit attempts compared to those with no previous quit attempts.

Both cigarette and waterpipe tobacco use rates reported in this study were lower than the ones reported in a recent household survey (Nakkash et al., 2022). This could be explained by the demographic characteristics of our study sample with 43% having university degrees. There is a well-established association between education and smoking behaviour with higher levels of education generally associated with lower rates of smoking (Maralani, 2014). Reasons for this association include the fact that higher levels of education lead to better health knowledge, attitudes, and behaviours, including a greater awareness of the risks of smoking and a greater ability to quit (Maralani, 2014, Ruokolainen et al., 2021, Holm et al., 2017).

The association between income and smoking behaviour is multifactorial and could have several explanations. Some studies have reported that people with higher income are more likely to have intentions to quit smoking and that people from low socio-economic groups face greater barriers to quitting as they are more likely to have stress related to material hardship, higher rates of smoking amongst family and friends and less access to smoking cessation support (Reid et al., 2010, Hiscock et al., 2015). However, in this study, we found that adults with a higher household income have lower intentions to quit cigarette smoking. Social acceptability and cultural norms surrounding smoking can play an important role in how individuals perceive the threat of smoking. In settings where smoking remains socially acceptable, such as Lebanon, people may be more likely to perceive smoking as less of a threat due to its widespread use and cultural acceptance (Nasser et al., 2020, Akl et al., 2015, Salloum et al., 2019).

Noteworthy, patterns of tobacco consumption have changed during the COVID-19 pandemic. A systematic review examining the impact of COVID-19 on smoking behaviour globally found that tobacco use decreased during the pandemic, with a majority of people who smoke reducing their cigarette and e-cigarette consumption compared to pre-pandemic levels (Almeda and Gómez-Gómez, 2022). This decline in smoking can be attributed to increased motivation to quit as a result of fear surrounding the pandemic and the negative consequences of COVID-19 (Almeda and Gómez-Gómez, 2022). The implementation of lockdown measures and reduced social activities also contributed to the decrease by limiting opportunities for social smoking and access to cigarettes (Almeda and Gómez-Gómez, 2022). However, certain regions, such as Iceland and specific parts of the United States, observed an increase in tobacco consumption during the pandemic. This rise may be associated with feelings of boredom during lockdown and the adoption of smoking as an unhealthy coping mechanism for stress (Almeda and Gómez-Gómez, 2022).

Research on the relationship between past quit attempts and intention to quit smoking in the Eastern Mediterranean Region is limited. However, studies conducted worldwide have shown a positive association between past attempts and intentions to quit smoking. A study conducted in China aiming to identify correlates of intentions to quit smoking among adults reported that past quitting attempts, their duration, and outcome expectancy of quitting were positively associated with intentions to quit smoking (Feng et al., 2010). Another study aiming to quantify the difficulty and intention to quit smoking in Switzerland reported that the intention to quit significantly increased twofold if the individual had 1 to 5 past attempts and by 4 times if the individual had 6 or more previous attempts (Marques-Vidal et al., 2011).

Having intentions to quit smoking is an essential factor in the process of quitting as it could be defined as an individual conscious plan or decision to quit which lies a stage before the action in the Transtheoretical Model. Several studies have shown that people having strong intentions to quit smoking are more likely to successfully quit compared to those with no intentions. In a cohort study conducted in Australia, Canada, the United Kingdom, and the United States to identify individual-level predictors of smoking cessation behaviour, intention to quit, a quit attempt in the previous year, and longer duration of past quit attempts were all predictive factors for making a quit attempt (Hyland et al., 2006).

This study has limitations. First, it was based on a telephone survey. A total of 6,068 calls were considered unsuccessful which may have resulted in non-response bias. Second, the proportion of respondents with a university degree was high (43% compared to 20% in the previous household survey (Nakkash et al., 2022), which suggests that the sample may be biased with limited representativeness. Third, smoking was self-reported which may have resulted in response bias. Finally, in the regression analysis, the small sample size of respondents with intentions to quit cigarette or waterpipe tobacco use resulted in wide confidence intervals, thus a larger margin of error. A larger sample size would have resulted in more accurate estimates.

In this study, the prevalence of tobacco smoking in Lebanon was estimated to be 41% (22% for cigarette and 20% for waterpipe) with 12% of adults who smoked having intentions to quit within the next 6 months. Past quit attempt(s) was considered a strong predictor of thinking of quitting both cigarette and waterpipe tobacco use. Given the social acceptability of smoking in the region, particularly in Lebanon, more research is needed to understand the factors that influence the association between past quit attempts and intentions to quit smoking. Understanding the effect of previous quit attempt failures, their timing, circumstances as well as individual factors such as motivation, self-efficacy and nicotine dependence in shaping this association can help inform the development of effective smoking cessation interventions.

## Funding

This study was funded by the International Development Research Centre (Grant number 108821).

## CRediT authorship contribution statement

**Dina Farran:** Data curation, Formal analysis, Methodology, Validation, Visualization, Writing – original draft. **Ruba Abla:** Data curation, Methodology, Project administration, Validation, Writing – review & editing. **Rima Nakkash:** Conceptualization, Funding acquisition, Methodology, Supervision, Validation, Writing – review & editing. **Niveen Abu Rmeileh:** Conceptualization, Writing – review & editing. **Mohammed Jawad:** Conceptualization, Writing – review & editing. **Yousef Khader:** Conceptualization, Writing – review & editing. **Aya Mostafa:** Conceptualization, Writing – review & editing. **Ramzi G. Salloum:** Conceptualization, Methodology, Supervision, Validation, Writing – review & editing. **Ali Chalak:** .

## Declaration of competing interest

The authors declare that they have no known competing financial interests or personal relationships that could have appeared to influence the work reported in this paper.

## Data Availability

Data will be made available on request.
